# Blockade of PI3K/AKT signaling pathway by Astragaloside IV attenuates ulcerative colitis via improving the intestinal epithelial barrier

**DOI:** 10.1186/s12967-024-05168-w

**Published:** 2024-04-30

**Authors:** Xinhui Zhang, Fan Zhang, Yan Li, Na Fan, Ke Zhao, Anding Zhang, Jiefang Kang, Yan Lin, Xiaochang Xue, Xun Jiang

**Affiliations:** 1 Department of Pediatrics, Tangdu Hospital, Air Force Medical University, Xinsi Road, Baqiao District, 710038 Xi’an, Shaanxi China; 2 The Key Laboratory of Medicinal Resources and Natural Pharmaceutical Chemistry, The Ministry of Education, College of Life Sciences, Shaanxi Normal University, 620 West Chang’an Avenue, Chang ’a District, 710119 Xi’an, Shaanxi China; 3 Medical College, Yan’an University, 580 ShengDi Road, Baota District, 716099 Yan’an, Shaanxi China; 4 Department of Nutrition and Health, China Agriculture University, 100091 Beijing, China

**Keywords:** Astragaloside IV (AS-IV), Ulcerative colitis (UC), Network pharmacology, Mucosal barrier, PI3K/AKT

## Abstract

**Background:**

The specific pathogenesis of UC is still unclear, but it has been clear that defects in intestinal barrier function play an important role in it. There is a temporary lack of specific drugs for clinical treatment. Astragaloside IV (AS-IV) is one of the main active ingredients extracted from Astragalus root and is a common Chinese herbal medicine for the treatment of gastrointestinal diseases. This study aimed to determine whether AS-IV has therapeutic value for DSS or LPS-induced intestinal epithelial barrier dysfunction in vivo and in vitro and its potential molecular mechanisms.

**Methods:**

The intestinal tissues from UC patients and colitis mice were collected, intestinal inflammation was observed by colonoscopy, and mucosal barrier function was measured by immunofluorescence staining. PI3K/AKT signaling pathway activator YS-49 and inhibitor LY-29 were administered to colitic mice to uncover the effect of this pathway on gut mucosal barrier modulation. Then, network pharmacology was used to screen Astragaloside IV (AS-IV), a core active component of the traditional Chinese medicine *Astragalus membranaceus*. The potential of AS-IV for intestinal barrier function repairment and UC treatment through blockade of the PI3K/AKT pathway was further confirmed by histopathological staining, FITC-dextran, transmission electron microscopy, ELISA, immunofluorescence, qRT-PCR, and western blotting. Finally, 16 S rRNA sequencing was performed to uncover whether AS-IV can ameliorate UC by regulating gut microbiota homeostasis.

**Results:**

Mucosal barrier function was significantly damaged in UC patients and murine colitis, and the activated PI3K/AKT signaling pathway was extensively involved. Both in vivo and vitro showed that the AS-IV-treated group significantly relieved inflammation and improved intestinal epithelial permeability by inhibiting the activation of the PI3K/AKT signaling pathway. In addition, microbiome data found that gut microbiota participates in AS-IV–mediated intestinal barrier recovery as well.

**Conclusions:**

Our study highlights that AS-IV exerts a protective effect on the integrality of the mucosal barrier in UC based on the PI3K/AKT pathway, and AS-IV may serve as a novel AKT inhibitor to provide a potential therapy for UC.

**Graphical abstract:**

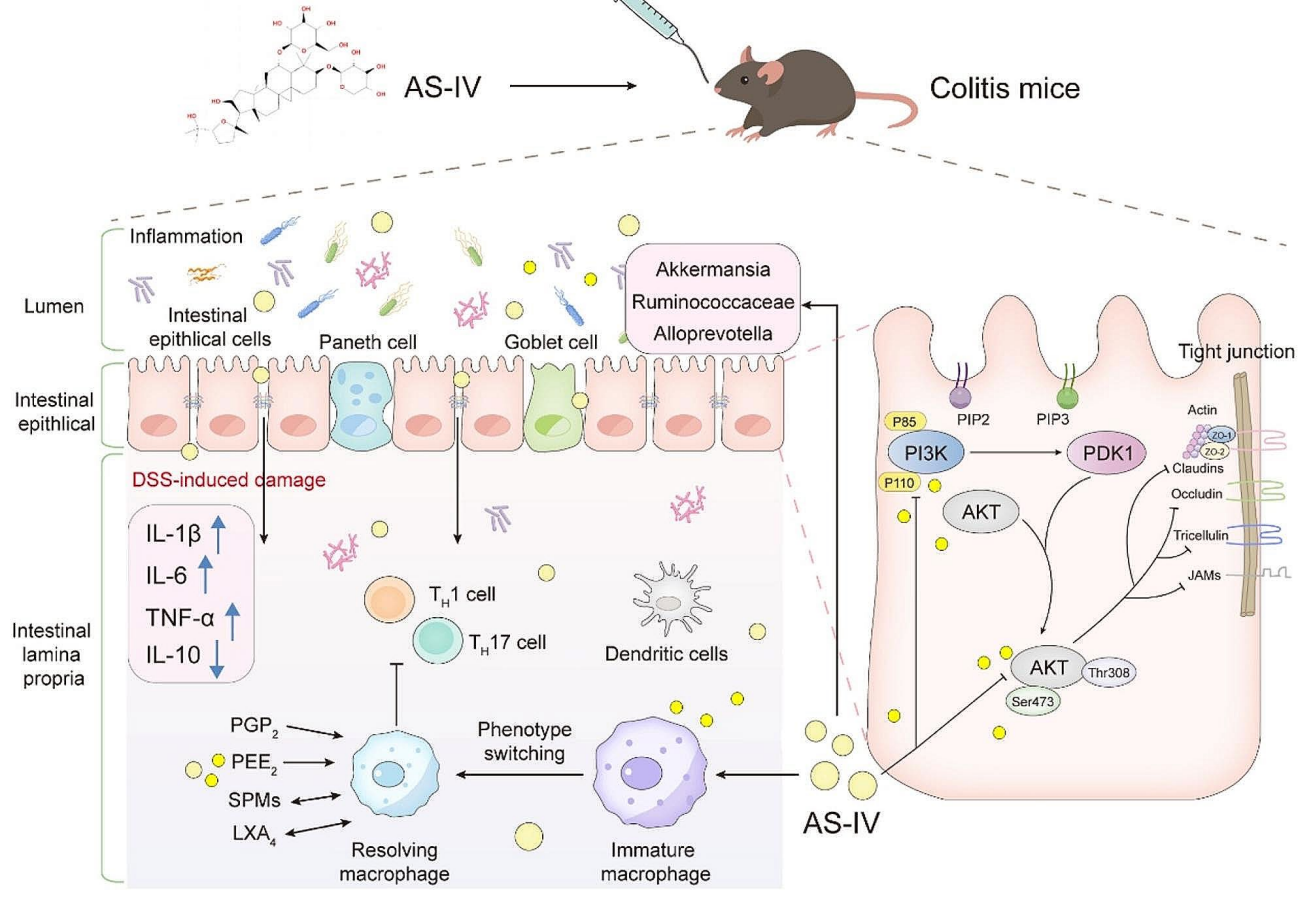

**Supplementary Information:**

The online version contains supplementary material available at 10.1186/s12967-024-05168-w.

## Introduction

Ulcerative colitis (UC) is a chronic non-specific inflammatory disease of the colorectum of unknown etiology, mostly located in the sigmoid colon and rectum, with lesions generally confined to the mucosal and submucosal layers. The occurrence of UC may be due to genetic predisposition, immune dysfunction, intestinal microecological dysregulation, and altered environmental factors, among others [1]. In the 21st century, UC has become one of the major challenges in global public health [2]. In Europe and North America, the number of patients exceeds 1.5 million and 2 million, respectively [3], and the prevalence in regions such as Eastern Europe, Asia, and Africa is continuously increasing [4].

Patients with UC tend to present with intestinal reactions with varying degrees of systemic symptoms, particularly disturbed bowel habits, including abdominal pain, diarrhea, and mucopurulent bloody stools. Although abdominal pain, diarrhea, and blood in stools are not life-threatening complications perse, they are associated with decreased quality of life, and the ensuing damage to the colonic mucosal barrier, and imbalance of intestinal flora are much more serious problems. The mucosal epithelium, as a natural protective barrier of the intestinal tract [5], not only regulates the selective permeability of the intestinal epithelial cells for material transport, but also prevents pathogenic antigens in the intestinal lumen, such as endotoxin, viruses, bacteria, and their metabolites, from entering into the intrinsic layer of the mucosa. When the intestinal tract is under an inflammatory state, the intestinal epithelial barrier will lose the normal transport and defense function, and harmful substances continuously enter the internal environment of the intestine, leading to excessive activation of the immune response, further damaging the intestinal barrier, and ultimately resulting in a series of inflammatory manifestations both inside and outside the intestine [6]. Based on the development of disease understanding, the current therapeutic goal for UC has changed from controlling inflammation and relieving clinical symptoms to promoting intestinal mucosal repair [7], a great goal that not only reduces the use of hormonal drugs and surgical treatments but also dramatically improves the long-term prognosis and enhances the quality of patients’ lives.

As above mentioned, dysregulation of the intestinal mucosal barrier is an important feature of UC disease, and this phenomenon is usually attributed to protein kinase B (AKT1), nuclear factor-κB (NF-κB), transcription factor 4 (TCF-4), mitogen-activated protein kinase (MAPK), and so on [8]. AKT is a protein kinase B with serine and threonine kinase activity, and its role is mainly related to the information linkage activated by phosphatidylinositol 3-kinases (PI3K) initiation [9]. The PI3K/AKT signaling pathway regulates the activation of inflammatory cells and the release of inflammatory mediators and has a clear intervention role in UC immune inflammation [10, 11]. NF-κB is an important intracellular nuclear transcription factor that participates in the body’s inflammatory response, and immune response, and regulates apoptotic and stress response. Christian F et al. found that the NF-κB signaling pathway in UC and colitis-associated colon cancer mice models was increased, which will lead to pathological changes in the UC intestine and trigger positive feedback for further NF-κB activation and exacerbated inflammation [12]. The Wnt pathway plays a crucial role in the intestinal epithelium, especially in regulating the behavior, proliferation, differentiation, and migration of stem cells [13]. Reduced Wnt pathway, especially the specific defect in TCF-4 expression induces deficiencies in Paneth cell defensins, which is one of the major factors in the pathogenesis of UC [14, 15]. The Notch signaling pathway is involved in the differentiation, proliferation, and apoptosis of colonic epithelial cells and promotes the expression of tight junction proteins. The aberrant activation of the Notch pathway induces an increase in the expression of the HES1 transcription factor in human colon cell lines, thereby inhibiting the differentiation of the secretory cell lineage and weakening the mucus barrier [16]. In addition, it also promotes inflammatory responses and accelerates the disruption of the intestinal mucosal barrier through activation of macrophage activation [17]. It is worth noting that in the above UC-related signaling pathway, we found that there are some important discrepancies seen in studies about the PI3K/AKT signal pathway. Firstly, while most studies suggest that PI3K/AKT signaling becomes overactive under colitis conditions [18–20], a few studies report reduced activities of PI3K/AKT in colitis animals [21, 22]. Secondly, it is in the preliminary research stage about clinically administrable chemical inhibitors and/or agonists for PI3K/ AKT at present. Thus, there is an urgent need to elucidate and validate the real function of the PI3K/AKT1 signal pathway in IBD.

Astragaloside IV (AS-IV) is a terpenoid extracted from *Astragalus membranaceus*, which is widely used in traditional Chinese medicine therapy and health care for its various pharmacological effects such as immunomodulation, anti-inflammation, antioxidant, anti-tumor, and hypoglycemic and lipid-lowering [23–25]. Accumulating evidence has demonstrated that AS-IV is extensively involved in the barrier function regulation of various diseases and the regulation of the PI3K/AKT pathway. AS-IV has been reported to have a significant protective effect on human glomerular endothelial cells (GEnCs) by inhibiting AKT activation and increasing GSK activation in the form of GSK3α, which promotes glucose uptake in GEnCs and enhances energy metabolism to participate in the protection of the filtration barrier in GEnCs [26]. AS-IV prevents tight junctions (TJs) disruption and intestinal barrier damage by inhibiting activation of RhoA/NLRP3 inflammatory vesicle signaling in an in vivo cecal ligation and puncture (CLP)-induced sepsis model and an in vitro Lipopolysaccharide (LPS)-primed Caco-2 monolayer barrier model [27]. AS-IV promotes the phosphorylation level of AKT, inhibits GSK-3β activity, leads to nuclear translocation and accumulation of β-catenin, improves cell viability, and promotes the expression of TJ proteins and wound healing to alleviate Peritoneal Dialysis (PD)-associated intestinal dysfunction [28]. Given that AS-IV plays an important role in the repair of barrier function in vivo, we sought to explore the potential impact of the administration of AS-IV on barrier function in dextran sodium sulfate (DSS)-induced colitis mice, and whether PI3K/AKT plays a role in it.

In this study about UC, we worked to investigate the regulation of the PI3K/AKT signaling pathway in intestinal inflammation, and the pharmacological activities of AS-IV such as anti-inflammatory and the repair of the intestinal epithelium. Simultaneously, we explored whether AS-IV acts as a potential ligand of PI3K/AKT. Finally, the potential microbial-dependent mechanism of AS-IV was also probed.

## Methods and materials

### Chemicals and reagents

Astragaloside IV was purchased from Dalian Meilun Co., Ltd. (Shanghai, China, purity > 98%). 5-amino salicylic acid (5-ASA) was purchased from MCE Ltd. (New Jersey, USA, purity ≥ 98%). DSS was purchased from MP Biomedicals (San Diego, USA). Sodium carboxymethyl cellulose (CMC-Na) and FITC-Dextran were obtained from Sigma-Aldrich (St. Louis, Missouri, USA). Interleukin (IL)-1β, tumor necrosis factor-α (TNF-α), and IL-6 enzyme-linked immunosorbent assay (ELISA) kits were from Shanghai Enzyme Union Co., Ltd. (Shanghai, China). β-actin antibody was purchased from Proteintech Co., Ltd. (Wuhan, China). Villin, zonula occludens-1 (ZO-1), Occludin, epithelial cell adhesion molecule (EpCAM), AKT1, p-AKT1, PI3K, and p-PI3K antibodies were from Abcam (Cambridge, UK) and CST Corporation (Boston, USA). Goat anti-rabbit IgG H&L (HRP) was obtained from Servicebio Co., Ltd. (Wuhan, China). Alexa Fluor 488-AffiniPure Goat Anti-Human IgG + IgM (H + L) was from Jackson Immuno Research (Pennsylvania, USA). LY294002 and YS49 were purchased from MCE Ltd. (New Jersey, USA).

### Human specimen acquisition

The intestinal mucosal tissue specimens of both UC patients and healthy control were obtained from Tangdu Hospital of Air Force Medical University and preserved according to the requirements of later experiments. Intestinal mucosal specimens were collected with the approval of the Ethics Committee of Tangdu Hospital, while patients were informed of the details and signed an informed consent form.

### Animals

C57BL/6 mice (male, 6–8 weeks old, 20–23 g body weight) were obtained from the Laboratory Animal Center of Air Force Medical University (Xi’an, China). Animals were acclimatized and fed for one week before the experiment under specific pathogen-free (SPF) conditions, with the temperature controlled at 22 ± 2 °C, while being given a 12-h light/dark cycle. All experiments involving animals complied with the regulations of the Ethics Committee for Animal Experiments of Air Force Medical University.

### Establishment and treatment of colitis murine model

The animal experiments in this study were divided into three parts: In the first part, mice were divided into two groups: control and DSS groups. The control group was given normal drinking water and the DSS group was given drinking water containing 2.5%DSS for 7 d. In the second part, mice were randomly divided into 4 groups: control, DSS, LY29, and YS49 groups. Drinking water containing 2.5%DSS was provided to the DSS and LY29 groups for 7 d, and the other two groups were given normal drinking water. During the period, LY29(5 mg/kg) and YS49(5 mg/kg) were injected intraperitoneally once into the LY29 or YS49 group every day respectively. In the third part, mice were divided into 4 groups: control, DSS, AS-IV treatment, and 5-ASA treatment groups. Except for the control group mice were provided with normal drinking water, the other groups of mice were given 2.5% DSS drinking water for 7 d. Mice in all groups were given normal drinking water from day 8 to day 10. All group mice gavage therapy was administered once a day from day 0 to 10. Mice in the AS-IV and 5-ASA treatment groups were gavaged 100 mg/kg AS-IV and 150 mg/kg 5-ASA respectively, and the control group and DSS group were gavaged 0.5% CMC-Na solution (solvent for AS-IV and 5-ASA). Each group of the three-part animal experiments contained 10 mice. We defined the starting point of the animal experiment as day 0 and recorded the weight and stool of the untreated mice. After the molding was done, all mice were anesthetized with 1% pentobarbital sodium (ip, 50 mg/kg) before sampling.

### Pharmacodynamic study

Body weight, diarrhea, and blood in stool were recorded at the same time each day during the modeling process, and the disease activity index (DAI) was scored [29]. At the end of the modeling period, feces and blood were collected from the mice. The colon was photographed and recorded, collected 1 cm from the end of the anus, and fixed with 4% paraformaldehyde, the remaining tissue was stored at -80 °C for RNA and protein extraction.

### Colonoscopy

Anesthetized mice with 1% pentobarbital sodium (ip, 50 mg/kg). Colonoscopy with a high-resolution mouse video endoscopic system (KARL STORZ, Tuttlingen, Germany) was used to inflate the colon with air and observe on mice’s colon.

### Permeability of FITC-dextran

After the animal model was completed, 3 mice were fetched randomly in each group. Every mouse was gavaged FITC-Dextran (40 mg/kg) after 8 h of fasting, and the abdomen was exposed carefully after 6 h to observe the distribution of FITC-Dextran in intestinal tissues using a small animal imaging system (Boloteng, Guangzhou, China). Meanwhile, the serum was separated to detect the fluorescence intensity after collecting the blood, and the concentration of FITC-Dextran in the serum was calculated by plotting a standard curve according to the instructions.

### Haematoxylin-eosin (H&E) and alcian blue-periodic acid Schiff (AB/PAS) staining

The colon of mice was obtained for pathological analysis. Colon tissues were fixed in 4% paraformaldehyde for 48 h and embedded in paraffin, sliced (thickness 5 μm), and attached to a high adhesion slide, numbered, overnight at 37℃. The section was stained with hematoxylin-eosin (H&E) [30] and alisin blue-periodic schiff (AB-PAS) [31].

### Transmission electron microscopy

Fresh colonic tissues were cut into 1-2mm^3^ blocks and fixed in glutaraldehyde fixative (Servicebio, Wuhan, China) for 2 h, resin embedding after alcohol gradient dehydration. Specimens were cut into semi-thin Sect. (0.5 μm) and ultrathin Sects. (70–90 nm) and observed using a transmission electron microscope (TEM 109, Zeiss, Jena, Germany).

### Cell culture and treatment

The human colorectal adenocarcinoma cell line (Caco-2) is similar in structure and function to differentiated small intestinal epithelial cells, with microvilli and enzymes associated with small intestinal brush epithelium, and can be used to simulate intestinal transport in vivo [32]. Caco-2 cells from ATCC/LGC Standards GmbH (HTB-37, Wesel, Germany) were cultured with MEM medium containing 20% fetal bovine serum (FBS) and 1% penicillin in a 37 °C, 5% CO_2_ cell culture incubator. AS-IV was dissolved in dimethyl sulfoxide (DMSO) for treatment of Caco-2 cells and the final concentration of DMSO was kept less than 0.1% (v/v). Caco-2 cells (1 × 10^4^ cells) were inoculated in 96-well plates and cultured at 37 °C, 5% CO_2_ to confluent, followed by stimulation with LPS (100 µg/ml) and AS-IV (150 µM) for 24 h. Then 10 µl/well CCK-8 (Yeasen Biotechnology, Shanghai, China) was added and incubated at 37 °C for 2 h further. Finally, absorbance at 450 nm was measured using a microplate reader (Thermo Fisher Scientific, Waltham, MA, USA).

### Immunofluorescence

Frozen sections of colons were fixed in acetone for 20 min and blocked with 10% goat serum for 1.5 h [33]. The Caco-2 cells crawling piece was fixed with 4% PFA [34]. After that, the slides were incubated with anti-ZO-1 (1:200), anti-occludin (1:250), or anti-villin antibody (1:100) overnight at 4 °C. Subsequently, sections were incubated with anti-mouse IgG (Alexa Fluor® 488 Conjugate) and anti-rat IgG (Cy3 Conjugate) in the dark at room temperature for 60 min, followed by incubation with 30 µL DAPI for 10 min. Finally, sections were observed using a fluorescence microscope (OLYMPUS, Tokyo, Japan).

### Real-time fluorescent quantitative PCR (qRT-PCR)

RNA was extracted from colon tissue and Caco-2 cells by TRIzol, and reverse transcription (Takara, Japan) and real-time PCR (Vayme, China) were performed according to the instructions of the kit. All primer sequences were synthesized by Sangon Biothen Co., Ltd. (Shanghai, China), and the primer sequences are shown in Table S1. All qRT-PCR analyses were carried out with a 7500 real-time PCR system (Applied Biosystems). The amplification protocol was as follows: 95 °C for 30 s, 40 cycles 95 °C for 10 s, 60 °C for 35 s, 95 °C for 15 s, 60 °C for 60 s, and 95 °C for 15 s. The relative fold change in expression was finally calculated using the 2^−ΔΔCT^ method. All samples were assayed in triplicate and expression levels of mRNA were normalized using the endogenous control β-actin [35].

### Enzyme-linked immunosorbent assay (ELISA)

Blood samples were collected from the mice and left for 1–2 h at room temperature. Then, 150–300 µl serum was obtained by centrifugation at 3500 rpm for 20 min. The culture medium of Caco-2 cells stimulated with LPS and AS-IV in a 12-well plate for 24 h was collected with a centrifuge at 12,000 rpm for 20 min. Cytokines including IL-1β, IL-6, or TNF-α were detected using ELISA kits (Shanghai Enzyme Union, Shanghai, China) in the serum or cell culture supernatant according to the manufacturer’s instructions. Capture antibodies (1:200) were coated in the plate overnight at 4℃. After blocking, samples were incubated for 2 h at room temperature, followed by incubation with detection antibodies (1:200) for 1 h. Subsequently, streptavidin conjugated with horseradish peroxidase was added, and the substrate was added 30 min later. Finally, the absorbance value was detected using a microplate reader. The concentrations of cytokines were obtained according to the standard curves.

### Western blot

Intestinal tissues and cells were collected and fully lysed with the pre-cooled RIPA mixture + 1% PMSF + 1% phosphatase inhibitor. The supernatant was collected with centrifugation. Protein concentration was then determined using BCA quantification (Thermo Scientific, Bremen, Germany), and heated at 100 °C for 10 min added 5x loading buffer. After being separated by electrophoresis using a 10% SDS-PAGE gel, the proteins were transferred onto a polyvinylidene difluoride (PVDF) membrane. Then, the PVDF membrane was blocked with 5% skimmed milk powder for 2 h at room temperature, and incubated with specific primary antibodies against EpCAM, Occludin, AKT1, p-AKT1(Ser473), PI3K, and p-PI3K (Tyr458) and secondary antibodies at 4 °C. Images were captured using a chemiluminescence imager (Tanon 5200, Shanghai, China), and quantitatively analyzed using the ImageJ software.

### Network pharmacology analysis

#### Molecular docking

AKT1 protein complex PDB file (PDB ID: 7NH5) from RCSB Protein Data Bank (https://www.rcsb.org/), the potential active site residues are obtained from the site finder module of the Molecular Operating Environment (MOE) software and protein plus (https://proteins.plus/). This operation establishes the structure-function relationship of the predicted AKT1 protein complex and identifies potential binding regions of antagonists or agonists. AS-IV (Compound CID: 13,943,297), Formononetin (Compound CID: 5,280,378), and Kaempferol (Compound CID: 5,280,863) structure information is from the PubChem database (https://pubchem.ncbi.nlm.nih.gov/). For the optimization of receptor and ligand molecules, hydrogenation and energy minimization are performed using MOE software. The DOCK module in MOE software is used for molecular docking, and the docking method is Induced Fi [36]. To eliminate false positive results, we restricted the docking score of AS-IV and AKT1 to less than − 5.0 kcal/mol [37]. Pymol was used to further display the docking results and analyze the interactions [38].

#### Screening of corresponding targets and establishment of disease target network

Relevant targets of AS-IV were collected from Traditional Chinese Medicine Systems Pharmacology Database (TCMSP, http://tcmspw.com/ tcmsp.php/) and Swiss target prediction databases (http://www.Swisstargetprediction.ch/) based on oral bioavailability (OB) ≥ 30% and drug-likeness (DL) ≥ 0.18. The Gene Cards database (https://www.genecards.org/) and Uniprot database (https://www.uniprot.org/) were used to search for UC-related target genes. Then, all the selected targets were imported into Cytoscape software to establish the active drug and disease target networks.

#### Construction of protein-protein interaction (PPI) network

To find the core targets for AS-IV intervention in UC, the STRING database (https://string-db.org/) was used to establish a protein-protein interaction (PPI) network of active drugs and common targets of diseases. The obtained data were imported into Venn diagram webtool (http://bioinformatics.psb.ugent.be/webtools/Venn/) and Cytoscape software to be analyzed and screened for possible core targets (the higher the degree indicates that the target is more important in this PPI network).

#### Analysis of gene ontology and KEGG pathway

To elucidate the possible molecular mechanisms of AS-IV for UC, we imported the common targets into the DAVID database (https://david.ncifcrf.gov/) and performed Gene Ontology (GO) and Kyoto Encyclopedia of Genes and Genomes (KEGG) enrichment analyses, biological process (BP), cellular component (CC) and molecular function (MF) were involved in it, and the results were filtered by *P* Value<0.05.

### 16 S ribosomal RNA (rRNA) gene sequencing

Colon fecal contents were collected, frozen with liquid nitrogen, and stored at -80°C. Total genomic DNA was extracted from fecal contents using QIAamp Fast DNA Stool Mini Kit (Qiagen, USA). Concentration of DNA was verified with NanoDrop 2000 (Thermo Fisher Scientific Co., Ltd.) and agarose gel electrophoresis. Then, 16S rRNA gene was amplified using hypervariable 3–4 (V3–V4) region-targeted barcoded universal bacterial primers, F: 5’-ACTCCTACGGGGAGGC AGCA-3’ and R: 5’-GGACTACHVGGGTWTCTAAT-3’ with the PCR system containing 25 µL of 2 × Premix Taq, 1.0 µL of each primer (10 µM), 50 ng of template DNA, and nuclease-free water added to 50 µL. PCR was conducted with an initial denaturation for 94 °C 5 min, 30 cycles at 94 °C 30 s, annealed at 52 °C 30 s, elongated at 72 °C 30 s, and a final extension at 72 °C 10 min on a thermocycler PCR system (Bio-Rad QX200 system, California, USA). The DNA library was built with NEBNext® UltraII DNA Library Prep Kit for Illumina (New England Biolabs, USA), and sequenced on the Illumina Nova 6000 platform (Guangzhou, China) in PE250 mode.

### Statistical analysis

Statistical analysis was performed using GraphPad Prism software (San Diego, USA) and is presented as mean ± standard error of the mean (SEM). The data were analyzed using a two-tailed Student’s t-test between two groups and a one-way analysis of variance followed by Dunnett’s post hoc tests when groups were more than two. *P* < 0.05 was considered statistically significant.

## Results

### Intestinal barrier integrity was severely destroyed in patients with UC and murine colitis

**Fig. 1:**
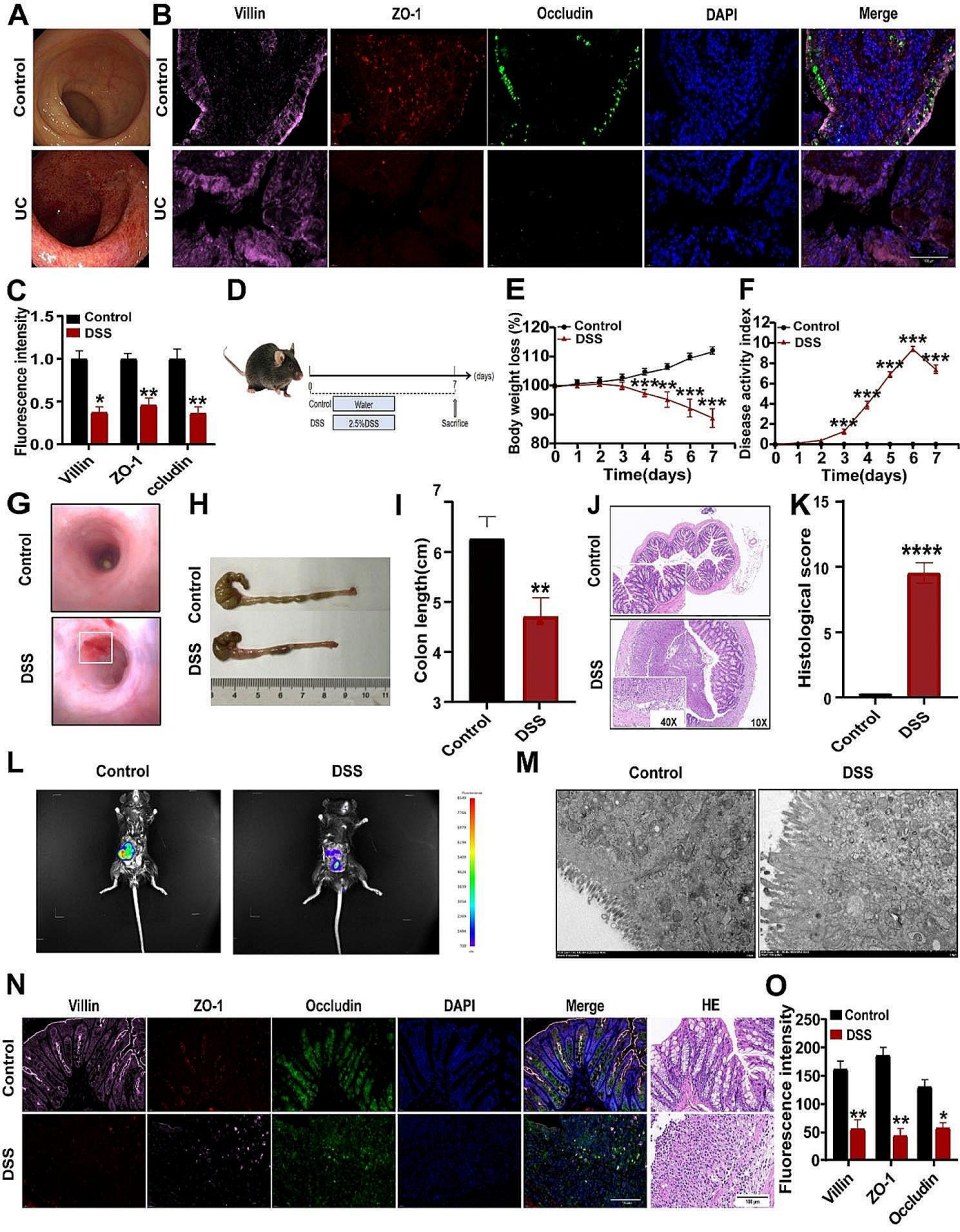
The integrity of the intestinal mucosal barrier is disrupted in UC patients and murine colitis. (**A**) Colonoscope observation of intestinal luminal mucosal tissue lesions in patients with UC. (**B**) Immunofluorescence was used to detect the expression of Villin, ZO-1, and Occludin in the colon tissues of healthy controls and patients with UC. Bar = 100 μm. (**C**) Expression of Villin, ZO-1, and Occludin in normal and diseased tissues by analyzing the data from (**B**) (*n* = 3). (**D**) Schematic diagram of the model induction process. (**E**) Body weight changes. (**F**) DAI scores. (**G**) Colonoscope observation of the colon tissues of normal and colitis mice. (**H**) Length of the colon. (**I**) Quantitative analysis of colon length. (**J**) HE staining of colon tissues. (**K**) HE staining score. (**L**) FITC-Dextran was administered to DSS-induced colitis mice and FITC-Dextran distribution in intestinal tissues was observed using a small animal imaging system. (**M**) Transmission electron microscopy observation of TJs. (**N**) Immunofluorescence detection of Villin, ZO-1, and Occludin expression in colon tissues of the mice. (**O**) Quantitative analysis of (**N**) Expression of Villin, ZO-1, and Occludin in the colon. bar = 100 μm. Data indicate the mean ± SEM. *P < 0.05 vs. the control. **P < 0.01, ***P < 0.001, ****P < 0.0001 vs. the control

The patients who were diagnosed with acute stage UC and had diarrhea, pain, and bloody stools were selected as study subjects. Colon mucosal congestion, edema with erosion, ulceration, bleeding spots on the intestinal mucosa, and the ulcer surface with white moss were observed under the colonoscope (the colon mucosa of healthy objects is smooth, with a clear vascular texture, and there is no erosion, ulceration, or bleeding) (Fig. 1A). Surgical resected colon tissues were collected from the subjects, and the intestinal barrier function was detected by immunofluorescence staining. Results showed that the expression of Villin, ZO-1, and Occludin, classical TJs-related proteins, on the inflamed colonic tissues was significantly decreased compared with that in normal control (Fig. 1B-C), indicating that gut barrier integrity is disrupted in the inflamed colonic tissues in UC patients. We wonder whether this phenomenon also exists in the murine colitis model. Therefore, C57BL/6 mice were fed with 2.5% DSS solution, and body weights, DAI scores, colon lengths, and colonoscope intestinal luminal manifestations were recorded and evaluated in conjunction with pathologic scores for the successful construction of a mouse model of colitis (Fig. 1D-J). Considering increased intestinal permeability is the main manifestation of mucosal barrier disruption and one of the basic features of UC [39], FITC-Dextran was orally administered to the colitic mice, and fluorescence intensity was detected in colonic tissues and sera. As shown in Fig. 1K, FITC-Dextran was widely distributed throughout the intestinal tissues up to the anus in the colitic mice as compared with the control mice. In addition, obvious rupture of the TJs structure of the epithelial cells and atrophy of the villi of the intestinal wall were observed under transmission electron microscopy (Fig. 1L). Consistently, the immunofluorescence results showed a significant reduction in the expression of intestinal TJs proteins Villin, ZO-1, and Occludin (Fig. 1M-N). These data collectively demonstrated that gut barrier integrity is disrupted and intestinal permeability is increased in the inflamed colonic tissues of UC patients and UC mice.

### PI3K/AKT signaling pathway activation increases intestinal barrier permeability

**Fig. 2:**
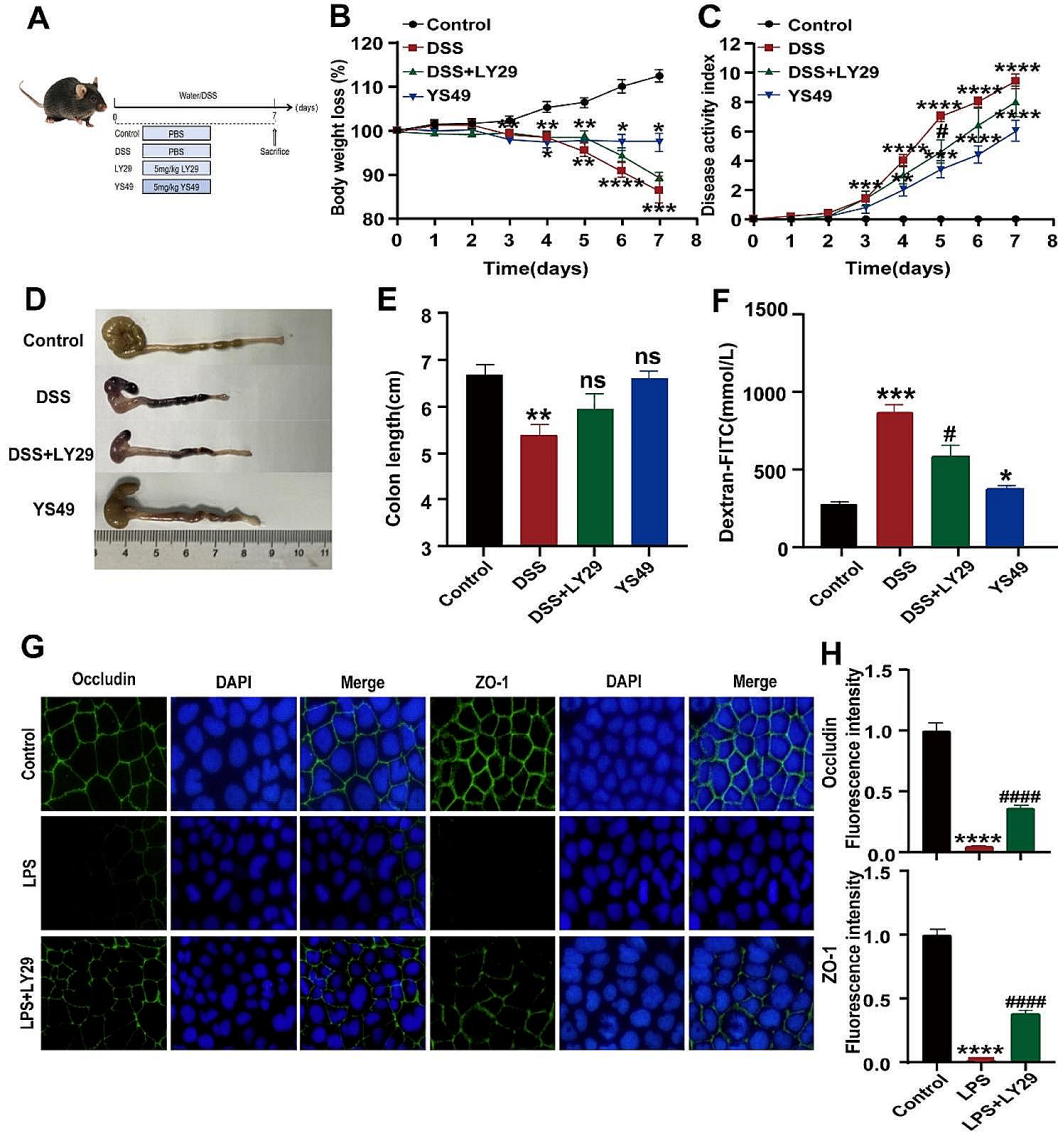
PI3K/AKT signaling pathway activation increases intestinal barrier permeability. (**A**) Schematic diagram of the model induction process. (**B**) Body weight changes. (**C**) DAI scores. (**D**) Length of the colon. (**E**) Quantitative analysis of colon length. (**F**) FITC-Dextran in serum. (**G**) Immunofluorescence of Occludin and ZO-1 expression in Caco-2 cells treated as indicated. (**H**). Quantitative analysis of (**G**). Data indicate the mean ± SEM. ns, not significant; *P < 0.05, **P < 0.01, ***P < 0.001, ****P < 0.0001 vs. the control; ^####^*P* < 0.0001 vs. the model group

As above mentioned, various signaling pathways have been reported to be extensively involved in UC occurrence and progression, and the PI3K/AKT axis participates in regulating the barrier function of various tissues in the human body, especially in intestinal barrier regulation [40, 41]. Whereas the conclusions are often contradictory, the real role and the detailed mechanisms of the PI3K/AKT axis in UC are still largely unknown. To clarify the effects of PI3K/AKT on UC, the activator YS49 is administered to normal mice, and the inhibitor LY29 is administered to DSS-induced colitis mice (Fig. 2A). As shown in Fig. 2B-F, YS-49 alone significantly reduced body weight (Fig. 2B), elevated DAI (Fig. 2C), shortened colon tissues (Fig. 2D-E), and increased intestinal permeability (Fig. 2F) of the C57BL/6 mice as compared with the control mice (*P* < 0.05), although not so significantly changed as DSS-treated mice (*P* < 0.01 or *P* < 0.001). Notably, LY29 remarkably inhibited DSS-induced colitis in mice as indicated by the recovered weight loss, colon length, DAI, and gut mucosa barrier. These data suggested that PI3K/AKT signal pathway activation damages intestinal barrier integrity and aggravates UC in mice. Then, we further confirmed this effect in intestinal epithelial-derived Caco-2 cells. Results showed that LPS stimulation greatly accelerated TJ disruption between Caco-2 cells via suppressing the expressions of ZO-1 and Occludin, which was potently restored in the presence of LY29 (Fig. 2G-H), indicating PI3K/AKT axis activation is essential for intestinal barrier damage.

### Network pharmacology predicts potential mechanisms for AS-IV treatment of UC

**Fig. 3:**
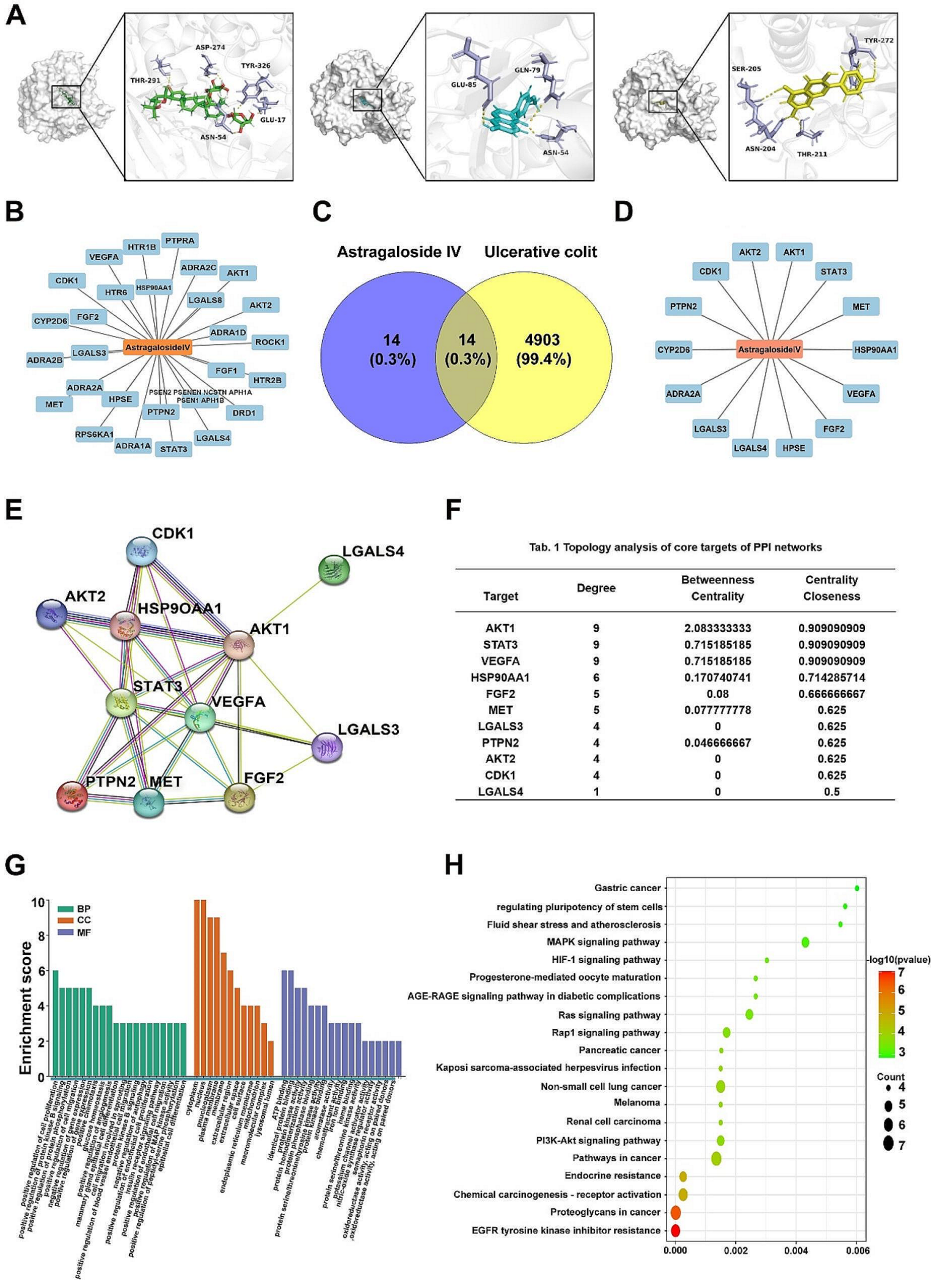
Network pharmacology predicts potential mechanisms for AS-IV treatment of UC. (**A**) Molecular docking modeling of AS-IV, Formononetin, and Kaempferol. (**B**) Corresponding targets of AS-IV collected by TCMSP and Swiss target prediction databases. (**C**) UC targets and AS-IV targets. (**D**) Co-acting targets of AS-IV and UC. (**E**) PPI network. (**F**) Analysis results of the PPI network. (**G**) GO enrichment analysis. (**H**) KEGG signaling pathway

Considering PI3K/AKT might be a potential target for UC treatment, molecular docking modeling was used to screen “AKT1 inhibitors” from dozens of compounds extracted from traditional Chinese medicine. Among them, AS-IV, kaempferol, and formononetin were found to have a relatively high affinity to AKT1. AS-IV is the most binding compound, the calculated binding energy is -12.596 kcal/mol, and four covalent bonds (Glu191, Lys276, Glu278, and Asp292) are possibly formed to tightly bind to AKT1 (Fig. 3A). Although we reported previously that AS-IV ameliorated DSS-induced colitis in mice via promoting macrophages M2 polarization [35], the direct target and detailed mechanism of AS-IV is almost completely unknown. Therefore, network pharmacology was used to explore the complex mechanisms of AS-IV in UC [42]. The TCMSP and Swiss target prediction databases were utilized to collect targets corresponding to AS-IV, and a total of 28 drug targets were obtained (Fig. 3B). Disease targets were screened using the Gene Cards database, and the relevance of the targets to the disease was evaluated by relevance score, where we screened a total of 4917 disease targets with relevance. Then the Venny data visualization tool was used to screen the drug-disease co-targets and draw Venny plots, and a total of 14 potential drug-disease combination targets were obtained (Fig. 3C-D). To further analyze the core targets of AS-IV in UC, the relevant data were imported into the STRING database and Cytoscape software to establish and analyze the PPI network, and the centrality of the nodes was analyzed by betweenness centrality (BC), closeness centrality (CC), and degree centrality (DC), and 11 meaningful targets were filtered out (Fig. 3E-F). Among them, AKT1 is the target gene with the highest number of nodes. Then KEGG and GO enrichment analysis of the above common targets was performed using the David database. Suggesting that AS-IV is involved in many biological processes in UC (Fig. 3G), including positive regulation of protein kinase B signaling and positive regulation of cell proliferation, the regulation of cellular components mainly focuses on the cytoplasm, nucleus, and nucleoplasm, and also plays a role by regulating molecular functions such as ATP binding, identical protein binding, and protein kinase activity. KEGG signaling pathway analysis showed that AS-IV was associated with EGFR tyrosine kinase inhibitor resistance, proteoglycans and pathways in cancer, chemical carcinogenesis-receptor activation, endocrine resistance, and PI3K/AKT signaling pathway was also highly correlated (Fig. 3H). PI3K/AKT signaling pathway (*p*-value = 1.40E-04, gene count = 6) was the most widely studied in UC and AKT1 was the key hub with the highest node degree in the PPI network. Combined with the above results and related literature hints [24], AS-IV may play a role in UC by regulating the signaling pathway PI3K/AKT.

### AS-IV attenuates inflammatory responses in murine colitis

**Fig. 4:**
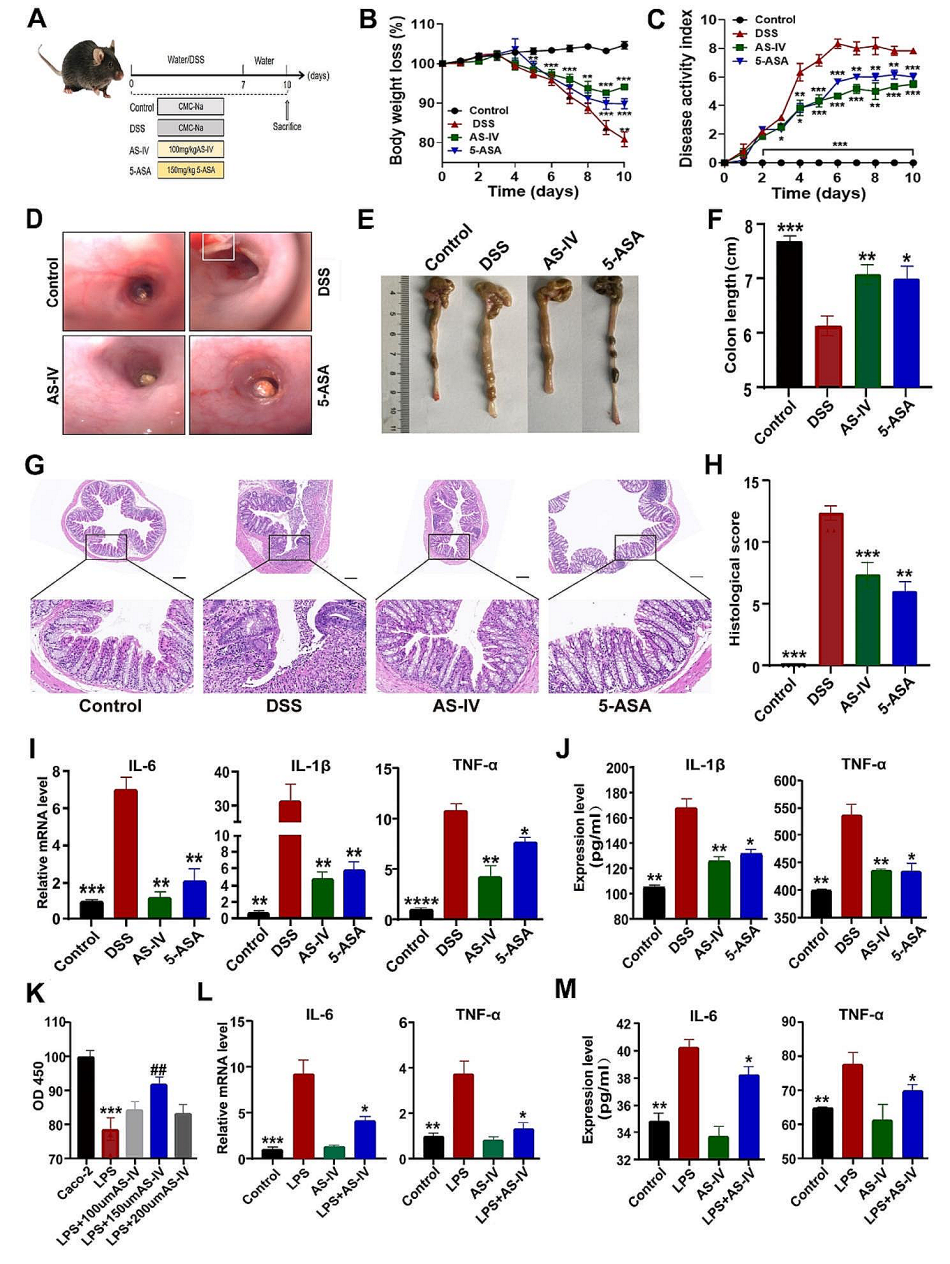
AS-IV attenuates inflammatory responses in murine colitis. (**A**) Schematic diagram of the model induction process. (**B**) Body weight changes. (**C**) DAI scores. (**D**) Colonoscope images of mouse colon tissues. (**E**) Length of colon. (**F**) Quantitative analysis of (**E**). (**G**) HE staining. (**H**) HE staining score. Relative mRNA levels of IL-6, IL-1β, and TNF-α in colon tissues were measured by qRT-PCR. (**J**) TNF-α and IL-1β expression levels in serum were detected by ELISA. (**K**) The effects of LPS and different concentrations of AS-IV on Caco-2 cell viability. (**L**) Relative IL-6 and TNF-α transcription levels in Caco-2 cells. (**M**) Relative IL-6 and TNF-α expression levels in culture medium. Data indicate the mean ± SEM. *P < 0.05, **P < 0.01, ***P < 0.001 vs. the DSS or LPS group for (**A**-**J**). ^##^*P* < 0.01 vs. the LPS group for (**K**)

As shown in Fig. 4A-C, E-H, the colitis mice after AS-IV intervention had less weight loss, lower DAI and pathology scores, and attenuated colonic length shortening as compared with the model mice. Meanwhile, the treatment of 5-ASA, which is identified as the routine medicine for IBD, was considered as the positive control. The AS-IV group showed smoother intestinal wall mucosa, improved congestion, and edema, and no obvious ulceration and bleeding spots compared to the DSS group under colonoscopy (Fig. 4D). In addition, both the mRNA and protein levels of proinflammatory cytokines IL-6, IL-1β, and TNF-α were significantly suppressed in colon tissues and sera (Fig. 4I-J). The effects of AS-IV on UC were further identified in an intestinal epithelial cell (IEC) inflammation model established by treating Caco-2 cells with LPS. As shown in Fig. 4 K, LPS stimulation significantly inhibited IEC viability, which was potently reversed in the presence of AS-IV. In addition, LPS induced strong proinflammatory cytokine IL-6 and TNF-α transcription (Fig. 3L) and expression (Fig. 3M) in IEC, while AS-IV treatment greatly suppressed this effect. Thus, AS-IV plays an anti-inflammation role in the murine colitis model.

### AS-IV regulates the intestinal barrier function in colitic mice

**Fig. 5:**
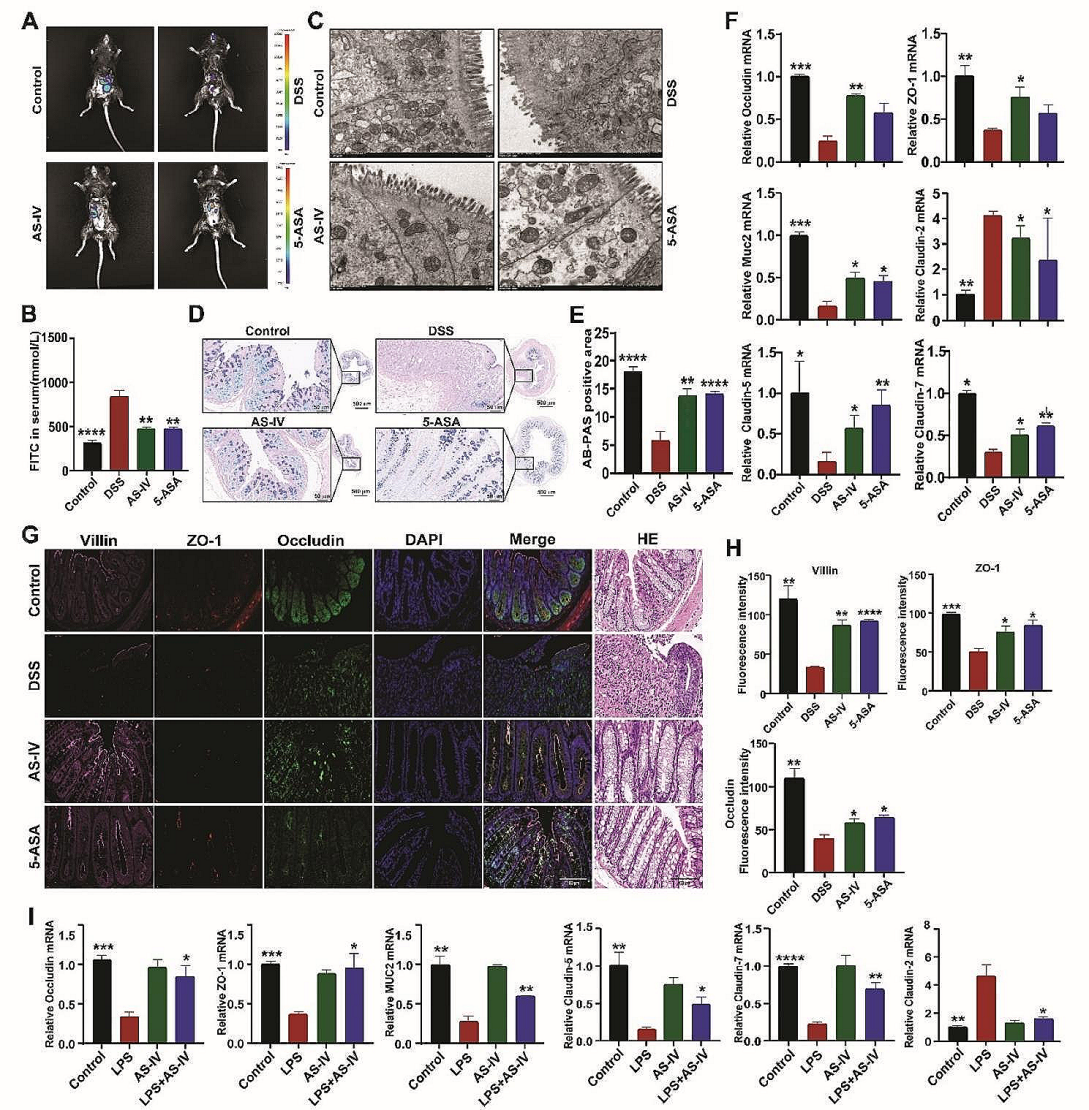
AS-IV regulates the intestinal barrier function in colitic mice. (**A**) FITC-Dextran in the colon was detected as previously described. (**B**) FITC-Dextran in serum. (**C**) Transmission electron microscopy observation of TJs. (**D**) AB-PAS staining. (**E**) Quantitative analysis of (**D**). (**F**) Relative expression of Occludin, ZO-1, MUC2, Claudin-2, Claudin-5, and Claudin-7 in colon tissues. (**G**) Immunofluorescence detection of Villin, ZO-1, and Occludin expression in colon. (**H**) Quantitative analysis of (**G**). (**I**) Relative expression of Occludin, ZO-1, MUC2, Claudin-5, Claudin-7, and Claudin-2 in Caco-2 cells treated as indicated. Data indicate the mean ± SEM. *P < 0.05, **P < 0.01, ***P < 0.001 vs. the DSS or LPS group

Subsequently, we assessed the effects of AS-IV on the intestinal permeability and mucosal barrier function in each group of the mice, results showed that the colons were significantly less permeable to FITC-Dextran after AS-IV intervention (Fig. 5A-B), and the disruption of the intestinal villi was alleviated under the transmission electron microscope, which was more neatly and densely arranged and tightly connected structures (Fig. 5C). AB-PAS staining was then performed on the colonic tissues of the mice, and results showed that AS-IV was able to alleviate the erosion of intestinal epithelium, crypt damage, and loss of mucus-secreting goblet cells induced by DSS (Fig. 5D-E). When the mRNA level of the TJs barrier-associated genes such as Occludin, Muc2, ZO-1, Villin, Claudin-2, Claudin-5, and Claudin-7 in the inflamed colonic tissues was detected by qRT-PCR, we found that the negative TJs regulatory gene Claudin-2 was suppressed, whereas the other positive TJs regulatory genes were significantly upregulated by AS-IV (Fig. 5F). In addition, immunofluorescence was performed to evaluate mucosal barrier TJs in the colon. Results showed that the expression of Villin, Occludin, and ZO-1 were all increased in the AS-IV group (Fig. 5G-H). These aforementioned data collectively suggested that AS-IV ameliorated murine colitis by improving the intestinal mucosa barrier function. Similar data were obtained in Caco-2 cells in which LPS stimulated significantly decreased, whereas AS-IV intervention increased the production of intestinal mucosa barrier-associated genes (Fig. 5I). Thus, AS-IV can directly regulate IEC-mediated gut barrier maintenance in the colitic mice.

### AS-IV inhibits PI3K/AKT activation in colitis

**Fig. 6:**
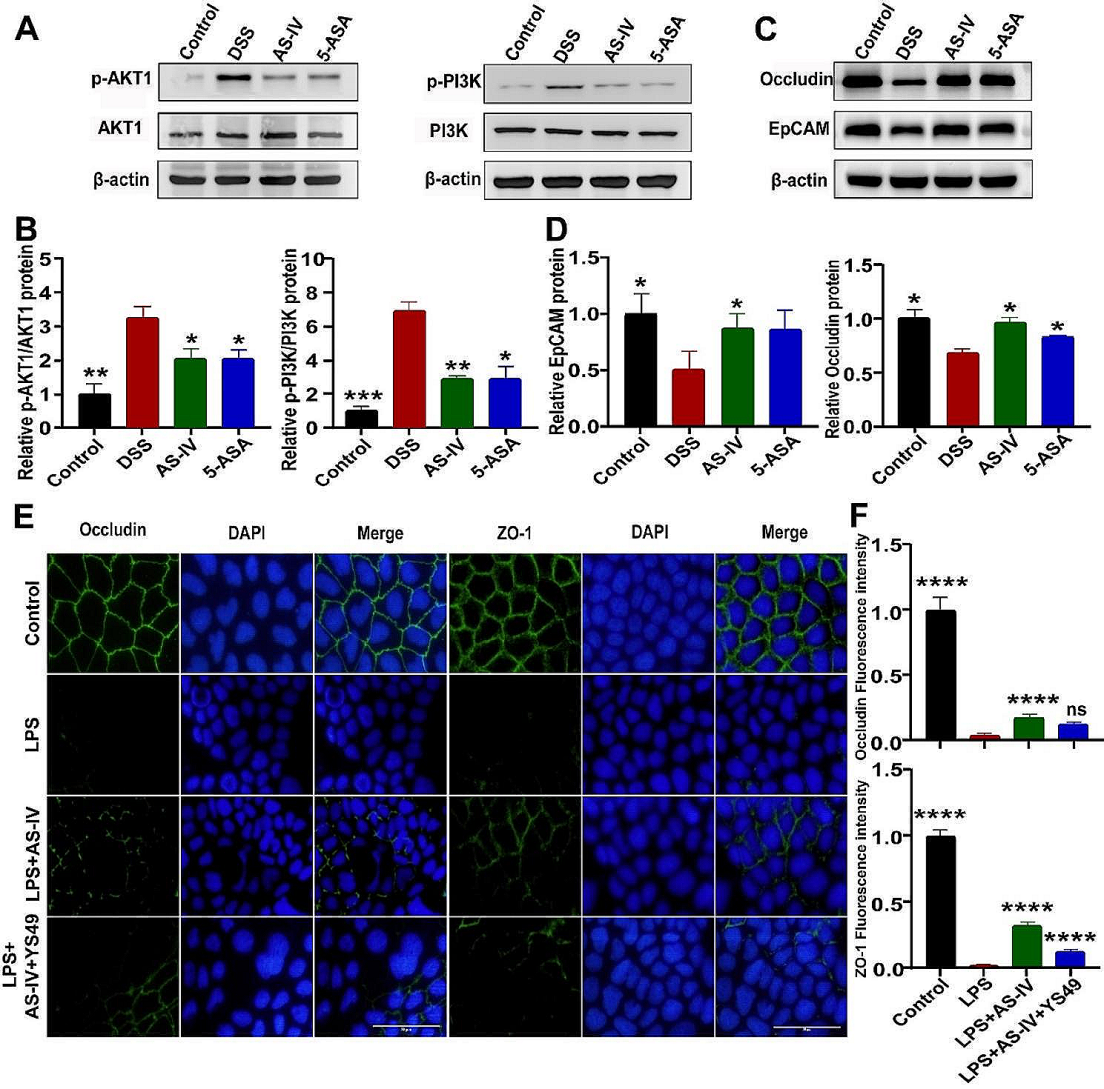
AS-IV inhibits PI3K/AKT activation in colitis. The levels of AKT1, p-AKT1, PI3K, and p-PI3K in colon tissues were measured by Western blot. (**B**) Quantitative analysis of (**A**). (**C**) Occludin and EpCAM expression in the colon tissues of the colitis mice was detected by Western blot. (**D**) Quantitative analysis of (**C**). (**E**) Occludin and ZO-1 expression in Caco-2 cells was determined by immunofluorescence. (**F**) Quantitative analysis of (**E**). Data indicates the mean ± SEM. ns, not significant; *P < 0.05, **P < 0.01, ***P < 0.001, ****P < 0.0001 vs. the DSS group

Although we predicted that AS-IV may directly target the PI3K/AKT signaling pathway, it is still unknown whether AS-IV regulated intestinal barrier function via this AKT molecule, and how about the effect of AS-IV on AKT activation. To clarify these problems, we detected the levels of PI3K, p-PI3K, AKT1, and p-AKT1 in colitis mice, and results showed that DSS stimulation strongly induced PI3K/AKT activation as indicated by the elevated phosphorylation of PI3K and AKT1. However, AS-IV significantly inhibited this effect (Fig. 6A-B). In addition, both the TJs-associated Occludin and adhesion junction-associated EpCAM were reversely changed (Fig. 6C-D), suggesting that AS-IV regulates the intestinal barrier via blocking PI3K/AKT activation. To confirm this, Caco-2 cells were treated as indicated and immunofluorescence was carried out to detect the changes of ZO-1 and Occludin, we found that LPS greatly inhibited ZO-1 and Occludin expression on Caco-2 cells, which can be restored by AS-IV. However, this effect of AS-IV was almost completely shielded by the PI3K/AKT activator YS-49 as exhibited by the downregulated ZO-1 and Occludin production (Fig. 6E-F). Therefore, we concluded that AS-IV regulates intestinal barrier maintenance in colitis via inhibiting PI3K/AKT activation.

### Gut microbiota participates in AS-IV–mediated intestinal barrier recovery

**Fig. 7:**
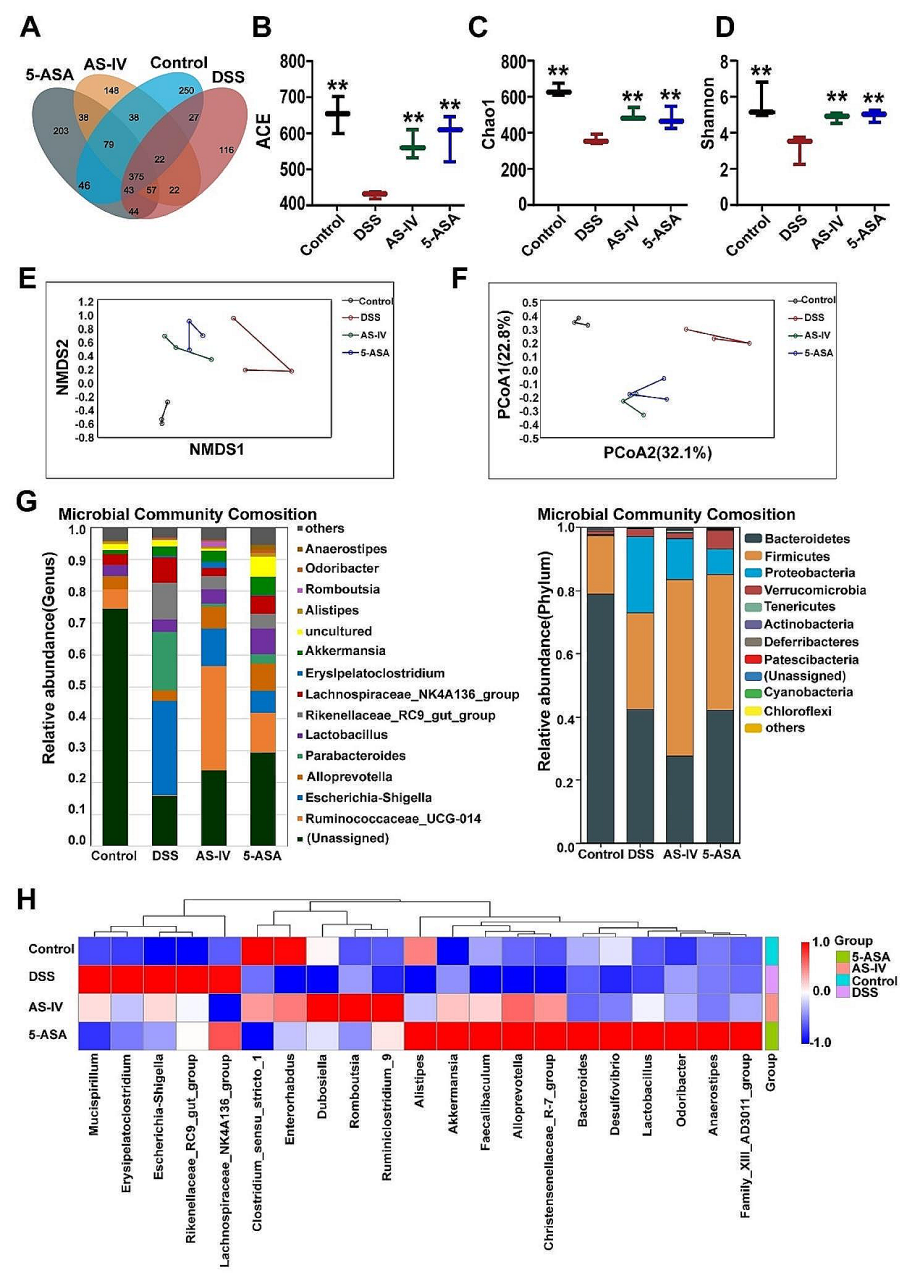
Gut microbiota participates in AS-IV–mediated intestinal barrier recovery. (**A**) Measurement of OTU. (**B**) ACE. (**C**) Chao1. (**D**) Shannon. (**E**) NMDS. (**F**) PCoA. (**G**) Species distribution at the phylum level (left panel) and genus level (right panel). (**H**) The heat map shows the cluster of species’ relative abundance. Data indicate the mean ± SEM, *n* = 3. *P < 0.05 vs. the DSS

Based on the therapeutic effect of AS-IV on colitis, we wonder whether AS-IV can regulate IECs and gut microbiota simultaneously as both of them play pivotal roles in colitis-associated inflammation and intestinal barrier disruption. Therefore, we analyzed the fecal samples by 16 S rRNA identification in the V3-V4 region, and the results showed that the bacterial species richness and diversity in feces in the AS-IV–-treated group were significantly increased compared to the control. The depth of sequencing of the OTUs of the Venny diagram covered all common species in the samples, and the overlap of the four groups represented the common OTUs, totaling 375, and group-specific OTUs of 203, 148, 250, and 116, respectively (Fig. 7A). In ecology, α diversity is commonly used to measure the diversity of intra-individuals [43]. By analyzing α diversity associated with OTU levels, such as Chao1, ACE, and Shannon indices, gut microbial was significantly reduced in the DSS group. However, the α diversity of the fecal microbial community in the AS-IV groups was restored after treatment with pharmacological interventions (Fig. 7B-D). Abundances of the taxa in all taxonomic ranks in each sample were used to construct sample-similarity matrices by the Bray-Curtis algorithm and analyzed the relative similarity of microbiota composition between groups according to non-metric multidimensional scaling (NMDS) and principal coordinate analysis (PCoA) [44]. As shown in Fig. 7E-F, the results of NMDS and PCoA showed that there were differences in microbial profiles among the four groups, with a relatively large distance between the control and DSS groups as indicated by a more unique microbiota composition, especially in terms of principal component 2 (PCoA2). Based on the results of the above analyses, we continued to investigate the microbial population composition at the phylum level, and most of the samples exhibited high levels of *Bacteroidetes*, *Firmicutes*, *Proteobacteria*, and *Verrucomicrobia* at the phylum level. DSS intervention significantly down-regulated the proportion of *Bacteroidetes* and increased the proportion of *proteobacteria* in the intestinal flora. However, the increase in the proportion of *firmicutes* was accompanied by a down-regulation of the proportion of *proteobacteria* in the AS-IV group (Fig. 7G). At the genus level, we observed *ruminococcaceae_UCG-014* (secreting butyrate), *Escherichia-Shigella* (producing the inflammatory factor IL-1β), *Alloprevotella* (producing short-chain fatty acids), and *Akkermansia* (the most promising next-generation probiotics) are as the dominant bacteria [45]. *Escherichia-Shigella* was increased by DSS but recovered after AS-IV treatment. In addition, the content of *ruminococcaceae_UCG-014*, *Alloprevotella*, and *Akkermansia* was significantly increased in the AS-IV group (Fig. 7H). It is known that *Akkermansia* is effective in increasing mucus thickness and strengthening intestinal barrier function [46]. The above results suggested that AS-IV plays a role in regulating the mucosal barrier by interfering with gut microflora homeostasis.

## Discussion

The elimination of intestinal inflammation is a complex biological process, involving multiple stages such as the repair of damaged intestinal mucosa and the regeneration of intestinal epithelial cells, and finally the termination of inflammation and the return of tissue homeostasis [47]. At present, the conventional treatment of UC mainly includes aminosalicylic acid, corticosteroids, and immunosuppressants, but these drugs are often accompanied by serious adverse effects and can even be life-threatening [48, 49]. It is worth noting that as one of the main active ingredients of Astragalus, AS-IV can exert biological effects by regulating the activity of proteins, thus inhibiting the overactivation of the immune response in vivo [24]. We demonstrated that the expression of intestinal mucosal epithelial TJs in UC patients and colitic mice was significantly decreased accompanied by an over-activated PI3K/AKT axis. However, AS-IV was able to effectively reduce the levels of pro-inflammatory factors, promote the expression of TJs barrier proteins, and ameliorate the damage to the intestinal mucosal barrier to alleviate murine colitis to a great extent. Meanwhile, 16 S rRNA sequencing showed that AS-IV significantly improved the intestinal flora disorders in colitic mice by increasing the proportion of *Firmicutes* decreasing the proportion of *Proteobacteria*, and significantly increasing the relative abundance of beneficial bacteria such as AKK. As the most promising next-generation probiotic, AKK can regulate glucose and lipid metabolism as well as mucus secretion, and limited studies have shown that AKK has the function of monitoring and regulating immune homeostasis [50] and can alleviate inflammation in IEC by down-regulating PI3K/AKT expression [51, 52]. These data collectively suggested that inhibiting the activated PI3K/AKT signaling pathway is a potential mechanism of AS-IV in UC treatment.

As a lipid kinase located near the inner layer of the plasma membrane, PI3K has Ser/Thr and phosphatidylinositol kinase activity. It is composed of a regulatory subunit, p85, and a catalytic subunit, p110, with the catalytic subunit having auto-suppressor properties, leading to near inactivation of the enzyme in the resting state [53, 54]. Upon activation, PI3K will phosphorylate phosphatidylinositol 4,5-bisphosphate (PIP2) to phosphatidylinositol 3,4,5-trisphosphate (PIP3) [55]. AKT is a subgroup of the Ser/Thr kinase family of AGC proteins that are a central node for cell signaling downstream of growth factors, cytokines, and other cellular stimuli. It can be activated with the help of the PI3K downstream effector phosphatidylinositol-dependent kinase 1 (PDK1) [56].

The activating of the PI3K/Akt signal pathway can induce NF-κB, LPS, and TNF-α-mediated pro-inflammatory and apoptotic signals by enhancing the phosphorylation of IκB (mainly IκBα) and reducing the synthesis of IκB, leading to the imbalance of cytokine secretion and enlarging the inflammatory chain reaction [57–59]. Eventually, inflammation and intestinal mucosal damage persist. American cockroach extracts Ento-A effectively increased the expression levels of IL-4 and forkhead transcription factor protein 3 (Foxp3) while decreasing the expression of interferon-γ (IFN-γ) and IL-17 in splenic lymphocytes, inhibited the PI3K/AKT/NF-κB signaling pathway, and ameliorated UC symptoms [19]. Hany et al. revealed that dapagliflozin exerts ameliorative effects on experimental colitis by inhibiting the HMGB1/RAGE/NF-κB cascade through activation of the AMPK/mTOR and Nrf2/HO-1 pathways, augmenting colon autophagy and inhibiting apoptosis [60]. Our study and the above literature have firmly supported that activated PI3K/AKT1 can lead to damage of intestinal mucosa by increasing the secretion of pro-inflammatory cytokines. Therefore, we have reason to think that the inhibition of PI3KAKT1 might be beneficial for the treatment of UC because it is more critical for the healing of mucosa to decrease the secretion of pro-inflammatory cytokines. The latter has been demonstrated to be a main factor directly causing mucosal damage.

Intestinal mucosal barriers mainly include mechanical, microbial, immune, and chemical barriers [61, 62], we mainly studied the mechanical and microbial barriers in this study. Our group previously reported that AS-IV can effectively inhibit pro-inflammatory macrophages and promote alternative macrophage polarization to improve the immune barrier of colitis by regulating the STAT signaling pathway [35]. These two studies provided a more comprehensive theoretical basis for the clinical research and application of AS-IV.

However, there are some shortcomings in this study. First of all, the use of Caco-2 cells as an in vitro intestinal barrier model instead of primary intestinal epithelial cells is a limitation. Currently, in vitro culture and processing of primary intestinal epithelial cells is still relatively difficult. Normally, organ cells gather together and adhere tightly to the extracellular matrix, and form a self-executing “home” that depends on the nutrient supply of growth factors for their function and survival. However, disturbance of the extracellular matrix or lacking growth factors usually leads to programmed cell death of IEC [63], thus greatly limiting the culture of intestinal epithelial primary cells in vitro. Secondly, genetically modified mice with mutant AKT1 which maintains its kinase activity but loses the AS-IV binding sites were not introduced is another limitation of this study. In future studies, we will attempt to be able to isolate and culture primary IECs from mouse intestines, obtain intestinal organoids, and build AKT1 mutant transgenic mice for in-depth exploration.

## Conclusions

In summary, we report here that AS-IV reduces inflammation and repairs the mucosal barrier of UC at least partially by blocking the activation of the PI3K/AKT signaling pathway. Our findings suggest that PI3K/AKT is a promising candidate target in UC therapy and provides new insights regarding the therapeutic potential of AS-IV for UC treatment, which has important research significance and clinical value.

### Electronic supplementary material

Below is the link to the electronic supplementary material.

Supplementary Material 1

## Data Availability

The authors do not have permission to share data. The data underlying this article are available in the article.

## Abbreviations

UC: Ulcerative colitis
AS-IV: Astragaloside IV
PKB: Protein kinase B
NF-κB: Nuclear factor kappa-B
AP-1: Activater protein-1
MAPK: Mitogen-activated protein kinases
TCF-4: Kinase transcription factor 4
GEnCs: Human glomerular endothelial cells
TJ: Tight junction
CLP: Cecal ligation and puncture
LPS: Lipopolysaccharide
PD: Peritoneal Dialysis
DSS: Dextran sulfate sodium
5-ASA: 5-amino salicylic acid
CMC-Na: Carboxymethyl cellulose
IL-1β: Interleukin-1β
TNF-α: Tumor necrosis factor-α
IL-6: Interleukin-6
ZO-1: Zonula occludens-1
IEC: Intestinal epithelial cell
SPF: Specific pathogen-free
DAI: Disease activity index
FBS: Fetal bovine serum
DMSO: Dimethyl sulfoxide
EpCAM: Epithelial cell adhesion molecule
MOE: Molecular Operating Environment
TCMSP: Traditional Chinese Medicine Systems Pharmacology
OB: Oral bioavailability
DL: Drug-likeness
PPI: Protein-protein interaction
GO: Gene Ontology
KEGG: Kyoto Encyclopedia of Genes and Genomes
BP: Biological process
CC: Cellular component
MF: Molecular function
BC: Betweenness centrality
CC: Closeness centrality
DC: Degree centrality
NMDS: Non-metric multidimensional scaling
PCoA: Principal coordinate analysis
AKK: Akkermansia
PIP2: Phosphorylate phosphatidylinositol 4,5-bisphosphate
PIP3: Phosphatidylinositol3,4,5-trisphosphate
PDK1: Phosphatidylinositol-dependent kinase 1
IFN-γ: Interferon-γ
