# Supplementary material for: Blockade of PI3K/AKT signaling pathway by Astragaloside IV attenuates ulcerative colitis via improving the intestinal epithelial barrier
Source: J Transl Med. 2024 Apr 30;22:406. doi: 10.1186/s12967-024-05168-w (PMC11061986; doi:10.1186/s12967-024-05168-w)
Supplement: Supplementary file 1 — Supplementary Material 1 [file 12967_2024_5168_MOESM1_ESM.docx]

**Supplementary Table. 1**

Human and mouse qRT-PCR primers

| Name | Tm(℃) | Primer sequences (5'-3') |
| --- | --- | --- |
| m-IL-6-F | 55.6 | GGCGGATCGGATGTTGTGAT |
| m-IL-6-R | 55.8 | GGACCCCAGACAATCGGTTG |
| m-IL-1β-F | 56.2 | CCTGAACTCAACTGTGAAATGCC |
| m- IL-1β-R | 56.2 | CAGCTTCTCCACAGCCACAATGAG |
| m-Actin-F | 50.01 | GGCTCCTAGCACCATGAAGA |
| m-Actin-R  m-Occludin-F  m-Occludin-R | 50.15  55.0  55.7 | ACTCCTGCTTGCTGATCCAC  TGAAAGTCCACCTCCTTACAGA  CCGGATAAAAAGAGTACGCTGG |
| m-ZO-1-F | 58.9 | GCCGCTAAGAGCACAGCAA |
| m-ZO-1-R  m-MUC2-F  m-MUC2-R  m-Claudin-7-F  m-Claudin-7-R  m-Claudin-5-F  m-Claudin-5-R  m-Claudin-2-F  m-Claudin-2-R | 54.7  59.01  60.24  61.0  61.4  67.81  67.95  65.65  64.87 | GCCCTCCTTTTAACACATCAGA  GCTGACGAGTGGTTGGTGAATG  GATGAGGTGGCAGACAGGAGAC  CCTGATAGCGAGCACTGCCATC  GTGACGCACTCCATCCAGAGC  GCTGGCGCTGGTGGCACTCTTTGT  GGCGAACCAGCAGAGCGGCAC  TGGCGTCCAACTGGTGGGCT  ACCGCCGTCACAATGCTGGC |
| h-IL-6-F | 59.05 | CAAAGATGTAGCCGCCCCAC |
| h-IL-6-R | 60.75 | GCCTCTTTGCTGCTTTCACAC |
| h-TNF-α-F | 57.08 | GAACTCACTGGGGCCTACA |
| h-TNF-α-R | 57.31 | GCTCCGTGTCTCAAGGAAGT |
| h-Actin-F | 55.26 | TGCAGAAAGAGATCACCGC |
| h-Actin-R  h-Occludin-F  h-Occludin-R | 55.82  54.99  53.73 | CCGATCCACACCGAGTATTTG  GCAAAGTGAATGACAAGCGG  CACAGGCGAAGTTAATGGAAG |
| h-ZO-1-F | 54.86 | CGAAGGAGTTGAGCAGGAAA |
| h-ZO-1-R  h-Muc2-F  h-Muc2-R  h-Claudin-7-F  h-Claudin-7-R  h-Claudin-5-F  h-Claudin-5-R  h-Claudin-2-F  h-Claudin-2-R | 54.64  61.10  57.92  53.68  54.14  69.07  51.51  54.84  58.54 | ACAGGCTTCAGGAACTTGAG  CTGCACCAAGACCGTCCTCATG  GCAAGGACTGAACAAAGACTCAGAC  TTCATCGTGGCAGGTCTT  AGGAACAGGAGAGCAGTG  ATGGGGTCCGCAGCGTTGGAGATCCT  GACGTAGTTCTTCTTGTCGT  GAGGGATTAGAGGTGTTCAAGG  AGGGACTGCTCCCTTGTCTT |
